# Exclusion of Exon 2 Is a Common mRNA Splice Variant of Primate Telomerase Reverse Transcriptases

**DOI:** 10.1371/journal.pone.0048016

**Published:** 2012-10-24

**Authors:** Johanna B. Withers, Tamara Ashvetiya, Karen L. Beemon

**Affiliations:** Department of Biology, The Johns Hopkins University, Baltimore, Maryland, United States of America; UMDNJ-New Jersey Medical School, United States of America

## Abstract

Telomeric sequences are added by an enzyme called telomerase that is made of two components: a catalytic protein called telomerase reverse transcriptase (TERT) and an integral RNA template (TR). Telomerase expression is tightly regulated at each step of gene expression, including alternative splicing of TERT mRNA. While over a dozen different alternative splicing events have been reported for human TERT mRNA, these were all in the 3′ half of the coding region. We were interested in examining splicing of the 5′ half of hTERT mRNA, especially since exon 2 is unusually large (1.3 kb). Internal mammalian exons are usually short, typically only 50 to 300 nucleotides, and most long internal exons are alternatively processed. We used quantitative RT-PCR and high-throughput sequencing data to examine the variety and quantity of mRNA species generated from the hTERT locus. We determined that there are approximately 20–40 molecules of hTERT mRNA per cell in the A431 human cell line. In addition, we describe an abundant, alternatively-spliced mRNA variant that excludes TERT exon 2 and was seen in other primates. This variant causes a frameshift and results in translation termination in exon 3, generating a 12 kDa polypeptide.

## Introduction

With each doubling of cultured human somatic cells, 50–200 base pairs of terminal DNA is lost [1], [2]. If the erosion of genetic material results in critically short telomeres, the cell signals senescence, apoptosis, or, in the case of a genetic aberration, genomic instability [3]. To circumvent the loss of essential genetic material, the ends of linear human chromosomes are capped with 5–15 kb of repetitive double-stranded telomeric sequence (TTAGGG) [4]–[7]. During S phase, telomeric sequences are added by a cellular reverse transcriptase known as telomerase that is minimally made of two components, a catalytic protein (hTERT) and an integral RNA template (hTR) [8]–[11].

In normal, non-dividing somatic cells, telomerase expression is very low or below detectable limits [12], [13]. This may allow telomeres to serve as a cellular checkpoint for aging [3]. Improper regulation of telomerase leads to uninhibited growth and cell division, the hallmarks of tumors [3], [6]. In order to prevent development of cancer phenotypes, telomerase is tightly regulated at each step of gene expression. In human cells, three main steps are minimally required to produce biologically functional telomerase RNP: accumulation of hTR, production of hTERT and assembly of catalytically active telomerase [14].

hTR RNA is present in 11,000–70,000 copies per cell in immortalized human cell cultures and is often detected even when telomerase catalytic activity is absent [13]. Therefore, absence of telomerase activity is most commonly correlated with a lack of functional hTERT protein. Ectopic expression of hTERT is often sufficient to restore telomerase activity [15]. The mRNA half-life of hTERT in cancer cells is estimated to be approximately 2 hours, while that of hTR is over 3 weeks [16], [17]. In addition to the more rapid decay of hTERT, production of functional wild type mRNA is further suppressed by alternative splicing. An estimated 95% of all hTERT mRNAs are alternatively spliced, and over a dozen different alternative splicing events have been reported [13], [18]. Almost all hTERT alternatively spliced variants reported to date disrupt the open reading frame and result in a premature termination codon that truncates the hTERT protein [18]. This also make the mRNAs likely targets of nonsense-mediated mRNA decay (NMD) [19].

**Figure 1:**
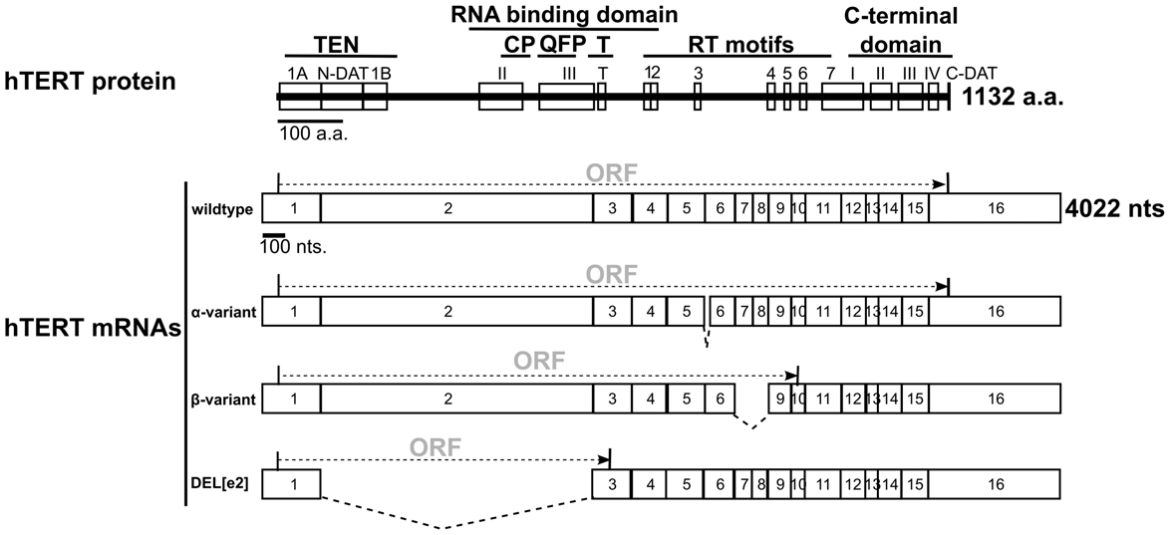
Molecular architecture of the hTERT coding region. Known hTERT protein domains and motifs are shown. The 1132-amino acid protein is aligned above the 3396-nt coding region of the 16 hTERT exons. Common alternatively spliced variants are shown below the wild type mRNA. These include the α-variant (deletion of first 36 nucleotides from exon 6), the ß-variant (deletion of exon 7 and 8) and the DEL[e2] variant (deletion of exon 2) described herein. The predicted open reading frame (ORF) for each mRNA is indicated. The 5′ UTR and 3′ UTR are not shown.

Wild type hTERT mRNA is generated by joining 16 exons (Figure 1). When we began this work, no alternative splicing events had been reported upstream of exon 4 [18]. We found this surprising because the second exon of hTERT is 1.3 kb, which is unusually large for an internal exon. In contrast, most internal mammalian exons are short, typically only 50 to 300 nucleotides [20], [21]. This narrow size distribution is governed by spliceosomal components that bind flanking sides of the exon at the splice site sequences to define exons and stimulate intron excision [20], [22], [23]. Less than 1.5% of internal exons are over 500 nts long, and most long internal exons are alternatively processed [24]. To gain a better understanding of hTERT alternative splicing, we used quantitative PCR and high-throughput sequencing data.

## Results

### Quantitative PCR of hTERT mRNA exon-exon junctions in human cells

**Figure 2:**
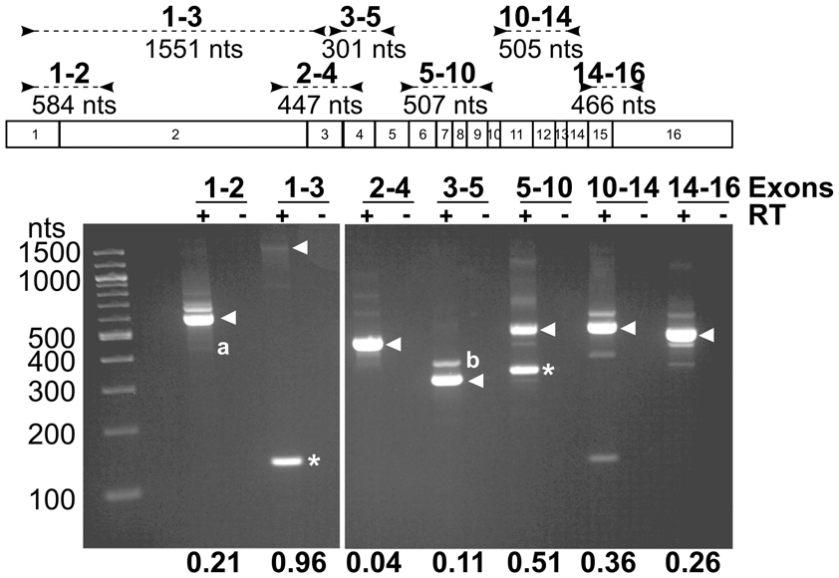
Deletion of exon 2 is a common alternative splice variant of hTERT mRNA. Amplified hTERT exon-exon junctions in an RT-PCR reaction of total RNA from A431 cells. hTERT exons, the location of each primer pair and the amplicon sizes predicted from wild type hTERT cDNA are indicated. The fraction of PCR products from alternatively spliced variants is shown below the gel. Wild type amplicons are represented by arrowheads, abundant alternatively spliced variants are indicated by an asterisk. Non-specific bands are indicated as “a” (actin bundling protein) and “b” (CDC27).

The low expression level of hTERT makes detection of the mRNA species difficult. We were unable to detect endogenous expression of hTERT mRNA by Northern Blot, primer-extension or RNase protection assays in human tumor cell lines (data not shown). Instead we initially relied on RT-PCR for detection of the alternatively spliced isoforms of hTERT. To detect hTERT mRNA in A431 cells (a human epidermoid carcinoma cell line), we used seven primer sets that collectively span all of the 15 exon-exon junctions present in the wild type mRNA (Figure 2). Multiple PCR reactions were prepared and stopped at increasing cycle numbers to ensure that the amplification was within the linear range (data not shown). Amplicons generated from a single PCR cycle for each primer pair are shown (Figure 2). At each splice junction, we observed an amplicon representing the wild type splicing pattern, which was confirmed by sequencing (Figure 2, arrowheads).

In addition to the wild type bands (Figure 2, arrowheads), we observed several alternatively spliced variants (Figure 2, *). Two of these were abundant, representing over 50% of the PCR product at that splice junction. The first is the well-known beta isoform that has been previously described and contains a deletion within the reverse transcriptase domain that removes exons 7 and 8 [18] (Figure 1). We observed 51% of the amplicons using primers in exons 5 and 10 were the beta isoform (Figure 2, primer pair 5–10; *). This splicing variant generates a premature termination codon in exon 10 and has no known function.

The second abundant alternative isoform observed was a deletion of the very large exon 2, DEL[e2] (Figure 2, primer pair 1–3; *). This deletion would generate a premature termination codon in exon 3. Despite the large amount of DEL[e2] mRNA amplification product that we observed (96% of the total PCR product) (Figure 2), we estimated that this alternative splice event occurred at a frequency that is approximately equal to the wild type splicing event after we corrected for the PCR amplification bias of shorter amplicons [26]. This novel alternative splice variant is not unique to A431 cells. We observed DEL[e2] transcripts by RT-PCR in every human cell line tested, including HeLa (cervical carcinoma), HEK293 (human embryonic kidney) cells and HCT116 (colon carcinoma) cells (data not shown).

With the primers in exons 1 and 2, we also observed a band larger than wild type that resulted from retention of intron 1 (Figure 2, primer pair 1–2). This band was called INS-i1 and contained 21% of the total PCR product. It would result in a premature termination codon 10 nucleotides into exon 2. We also observed retention of the first 349 nts of intron 2 to generate INS-i2 (Figure 2, primers 2–4).

### 20–40 hTERT mRNAs per cell estimated by competitive PCR

**Figure 3:**
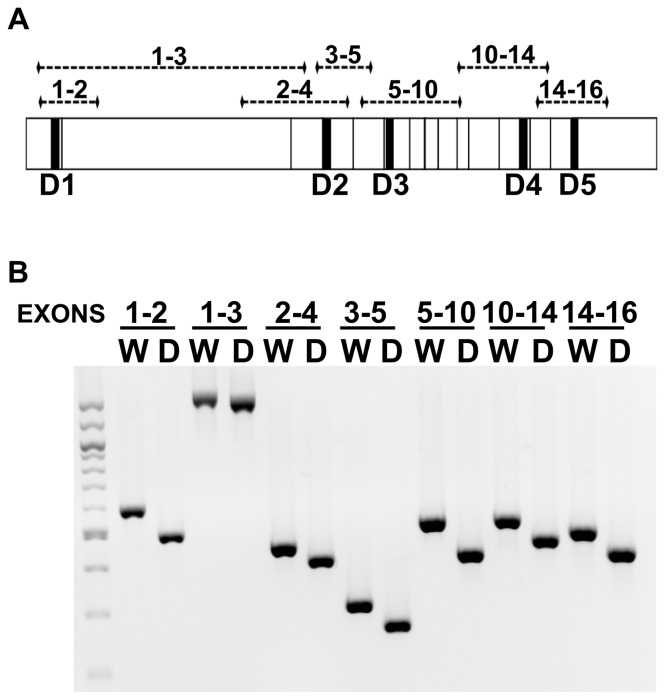
A competitive deletion hTERT construct results in amplicons that are approximately 10% smaller than wild type. A. Competitive PCR cDNA construct was generated by deleting 5 regions (D1–D5, black boxes) from wild type hTERT cDNA plasmid. Approximately 10% of each amplicon was deleted. B. PCR amplicons of plasmid DNA from wild type hTERT (W) and the competitive deletion hTERT construct (D) resolved on a 2% agarose gel.

Quantitative real-time RT-PCR is typically used to determine the relative level of mRNAs in the cell. While powerful, the accuracy of this technique relies on amplification of a single amplicon from an individual primer pair. When each primer pair recognizes multiple splice isoforms, real-time RT-PCR is no longer an effective method to analyze mRNA levels. Therefore, to accurately quantify hTERT mRNA isoforms levels, competitive PCR [27] was used. For this technique, a cDNA was generated from the wild type hTERT mRNA that contained five deletions within the amplified regions of interest. This allowed a single competitive cDNA to be used with all seven primer pairs (Figure 3A). The amplicons generated from the deletion cDNA could be readily separated from the wild type amplicons for each primer pair (Figure 3B).

**Figure 4:**
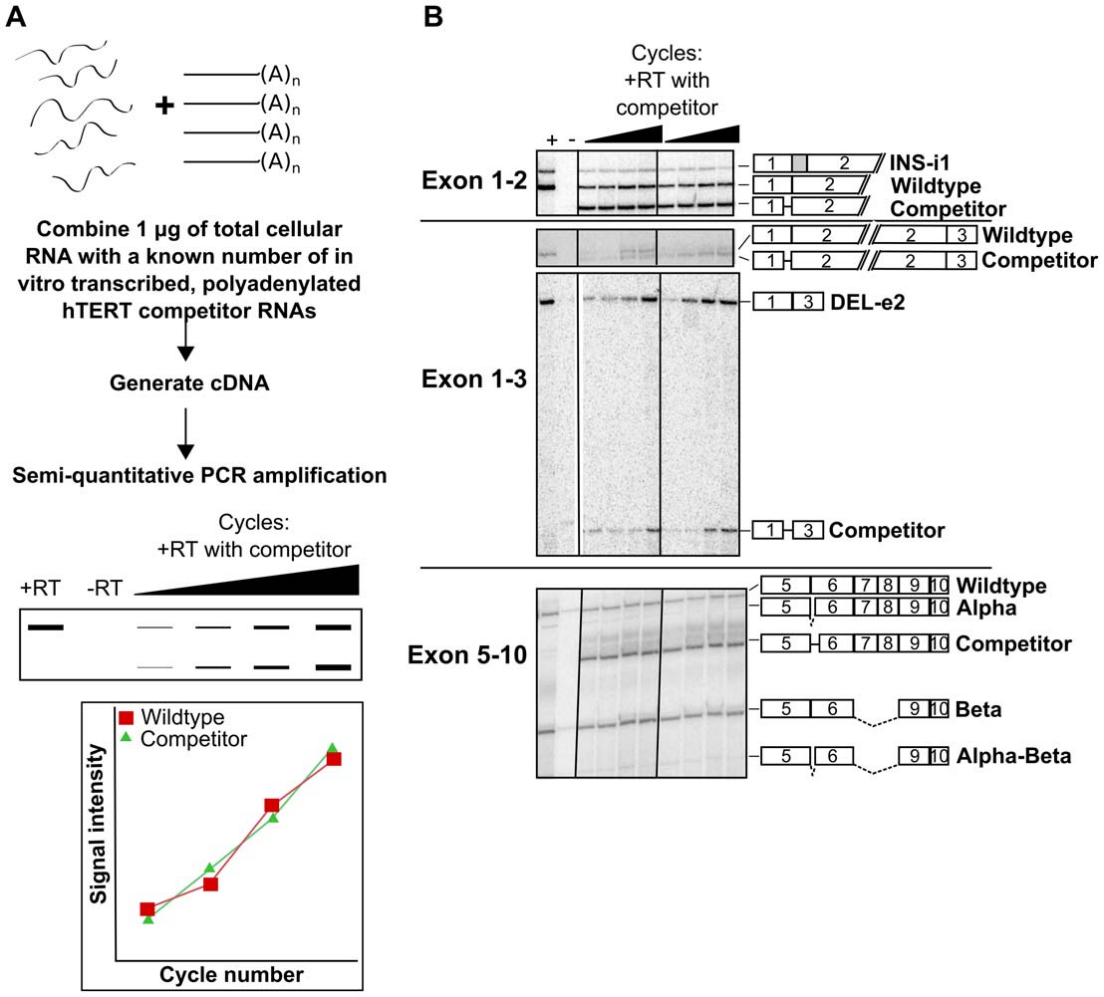
There are approximately 20–40 molecules of hTERT mRNA per cell. A. The *in vitro* transcribed deletion hTERT RNA was added to total cellular mRNA from A431 cells in varying amounts to generate a titration curve between 1×10^4^ and 1×10^7^ molecules of competitive RNA. This RNA sample was reverse transcribed so that both competitor and endogenous hTERT could be amplified in the same PCR reaction to determine the initial amount of hTERT mRNA present in the reaction. B. The forward primer was radiolabeled and hTERT RT-PCR of total A431 RNA was performed in the presence of hTERT competitive deletion constructs. Samples were separated on a 4% denaturing acrylamide sequencing gel. The identity of each band is indicated by the exon structure to the right. The amplicon is indicated by the dotted line above the diagrams. Data are representative of at least two independent experiments. DEL, deletion; INS, insertion; e, exon; i, intron.

This deletion cDNA was *in vitro* transcribed, polyadenylated and carefully quantified. The deletion RNA was mixed (in varying amounts ranging from 1×10^4^ to 1×10^7^ molecules) with a known amount of total cellular mRNA to generate a titration curve. The cellular RNA and competitive deletion RNA were reverse transcribed together and both were subsequently amplified in the same PCR reaction (Figure 4A). When the two were amplified at equivalent rates and levels (Figure 4A, bottom panel), the amount of competitive RNA present was used to determine the initial amount of hTERT mRNA present in the reaction. For each amount of competitive RNA tested, four identical PCR reactions were run, stopped every two cycles and analyzed on a denaturing acrylamide gel (Figure 4B and data not shown). One primer from each reaction was radioactively end labeled to enable quantitation that was independent of size.

To determine the amount of RNA derived from each cell, A431 cells were counted prior to harvesting total RNA. The total amount of RNA remaining after DNase treatment was divided by the original cell number. For the protocol used during these experiments, we obtained 0.036±0.05 µg of total RNA per 1000 A431 cells. The radioactive signal of each PCR product was quantified and normalized relative to the amplicon derived from the competitive RNA (Figure 4). Because the size of the deletion construct differed by only approximately 10% from the wild type band in each reaction, the size bias during PCR amplification was substantially reduced.

When normalized to the amount of the competitor and the amount of RNA in each PCR reaction, the amount of total hTERT mRNA was calculated to be approximately 20–40 mRNAs per cell (Table 1). For each primer pair, “wildtype” refers to the amplicon containing all annotated exons. We were also able to determine the approximate level of 15 alternative mRNA variants (Table 1). Many of the splice variants were expressed at levels that varied between the human cell lines examined. If approximately 95% of hTERT mRNAs are alternatively spliced for at least one junction [13], we estimate that there are approximately 1–2 mRNAs per cell capable of generating wild type hTERT protein. While this seems to be a low level, telomerase activity is only required during S phase at short telomeres [28], [29].

**Table 1:** Quantification of hTERT mRNA variants by competitive RT-PCR.

Exons	Construct	Molecules per cell
**1–2**	INS-i1	2.5±2.2
	Wildtype	10±3.8
**1–3**	Wildtype	17±6.0
	DEL-e2	19±6.4
**2–4**	INS-i2[1–349]	4.5±3.7
	Wildtype	18.5±15.6
**3–5**	Wildtype	22±7.9
**4–10**	Wildtype	4.5±1.3
	Alpha	1.25±1.1
	Beta	5.5±1.5
	Alpha-Beta	0.65±0.5
**9–14**	INS-i13	1±0.3
	Wildtype	5±1.3
**13–16**	INS-i14	2±0.5
	INS-i14[619–781]*	4±1.0
	INS-i14[622–705]*	3±1.6
	Wildtype	5.5±1.5

* Residue numbers inserted from intron 14 are indicated in brackets, with the first nucleotide of intron 14 denoted as “1”.

Interestingly, both the DEL[e2] variant and the wild type splicing pattern between exon 1 and 3 were detectable at nearly 20 molecules per cell in A431 cells. Thus, this novel DEL[e2] is the most abundant hTERT splicing variant in these cells. For comparison, the beta construct was present at about 5 copies per cell and alpha was present at 1 copy. In addition, the novel INS-i1 and INS-i2 variants were present at 2 and 4 copies per cell, respectively.

### Analysis of alternative hTERT exon 2 inclusion from deep sequencing databases

To gain a better understanding of the prevalence of the hTERT DEL[e2] alternative splicing event, we analyzed deep sequencing data from 120 publicly available datasets in the NCBI Sequence Read Archive. Deep sequencing reads aligned to a single mRNA were compared to the overall number of reads from a single sequencing experiment to determine the relative level of expression. When these reads aligned to an exon-exon junction, they could be assembled to identify alternative mRNA splice forms.

Due to the low expression level of hTERT, many datasets lacked reads corresponding to hTERT. Of the 120 datasets analyzed, only 78 contained sufficient hTERT-specific reads to proceed with our analysis. These included a large number of different human immortalized cell lines (including many lymphomas and carcinomas) as well as primary human tissues from brain, liver, heart, muscle, thymus, kidney, eye, ovaries and testis. To determine the frequency of exon 2 exclusion, we calculated the ratio of reads that mapped to exon 2 relative to exon 1 or exon 3, whichever had the highest expression, in each dataset as previously described [24]. Most often, exon 3 had more reads than exon 1.

**Figure 5:**
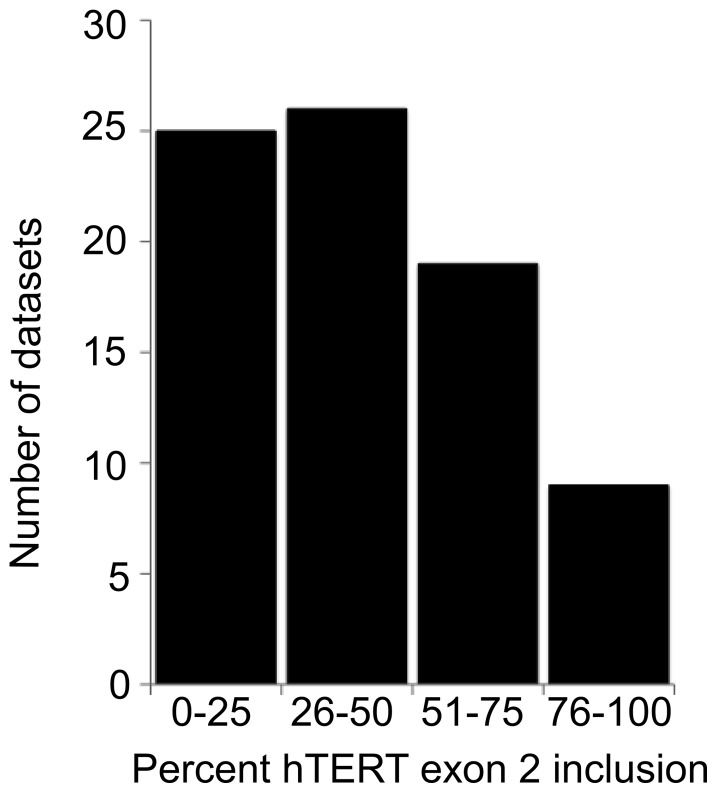
hTERT exon 2 is underrepresented relative to the flanking exons in deep sequencing datasets. The percent inclusion of hTERT exon 2, relative to either exon 1 or exon 3 (whichever was higher) was determined from 78 deep sequencing datasets. Datasets were binned into inclusion rates of 0–25%, 26–50%, 51–75% and 76–100%. TERT from ES cells contained high levels of exon 2, in contrast to other cell lines and primary tissues.

The hTERT DEL[e2] alternatively spliced variant was prevalent in almost all cell types analyzed that expressed hTERT. Exon 2 was present in 0–75% of the transcripts in most cell types analyzed, with a mean of 50% inclusion (Figure 5). Embryonic stem cells were an exception to this pattern; hTERT exons 1, 2 and 3 appeared to be expressed at equivalent levels, suggesting exon 2 is constitutively retained in this cell type.

### TERT alternatively spliced variants in metazoan species

**Figure 6:**
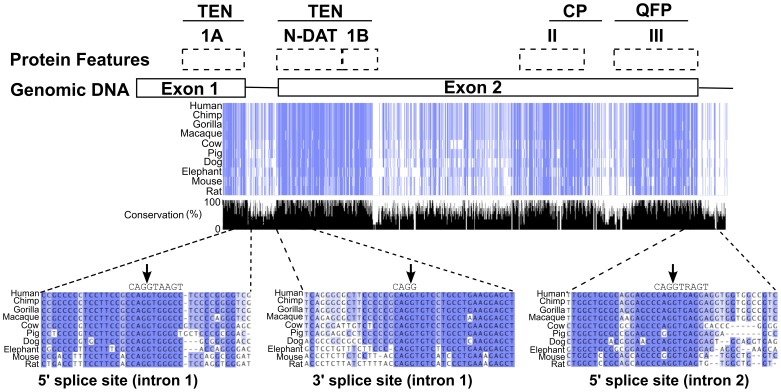
Large internal exon 2 is conserved in mammals. Genomic nucleotide sequences from the end of exon 1 to the beginning of intron 2 for human, chimpanzee, gorilla, macaque, cow, pig, dog, elephant, mouse and rat were aligned using Jalview. The locations of the human protein domains encoded by these nucleotide sequences are indicated above. Nucleotide residues are colored according to the percentage of residues in each column that are identical to the human sequence (>80% mid blue, >60% light blue, >40 light grey, <40% white). Only those residues that match the human sequence are colored. The percent conservation at each nucleotide position across all of the species listed is also indicated in the bar graph in black at the bottom. Conservation is calculated according to the Jalview and AMAS methods. Exons, and specifically the protein motifs and domains, are more conserved than flanking regions. 20 nucleotides flanking the exon junctions are shown in detail. The splice site consensus sequences are indicated above each highlighted region and the exon-exon boundaries are marked with arrows.

The large internal exon 2 in TERT is not unique to humans; it is present in many species, including primates, dogs, cows, pigs, elephants and rodents. Thus, we were able to align the genomic nucleotide sequences, from the middle of exon 1 to the beginning of intron 2, of 10 species that contain a large TERT exon 2 (Figure 6). The human protein domains are indicated above the sequences. These protein-coding sequences were well conserved at the nucleotide level, as indicated by the regions highlighted in blue. The N-terminal linker region that separates the TEN 1B domain from the CP domain was less conserved; this linker region varies in length between species and is largely insensitive to mutations and has little predicted secondary structure [30].

From this sequence analysis, the splice donors and acceptors of exons 1 and 2 appear to be well conserved between species and to align well with the splice site consensus sequences (Figure 6). However, splice site consensus sequences are often not sufficient to determine whether an exon will be included. Many RNA sequences can interact with splicing enhancers and silencers to coordinate spliceosome assembly [31], [32]. In fact, hTERT exon 2 had a very low ratio of exon splicing enhancers/exon splicing silencers of 1.3 (determined as described in [24]) that may contribute to its frequent exclusion. In contrast, constitutively spliced large internal human exons (>1 kb) had an average enhancer/silencer ratio of 4.25, and alternatively spliced large exons had an average ratio of 3.4 [24]. In addition, hTERT exon 2 contained 30 large exon splicing enhancer sequences (LESEs); this is slightly below the average of 26 LESE hexamers per kb in all human large internal exons [24].

**Figure 7:**
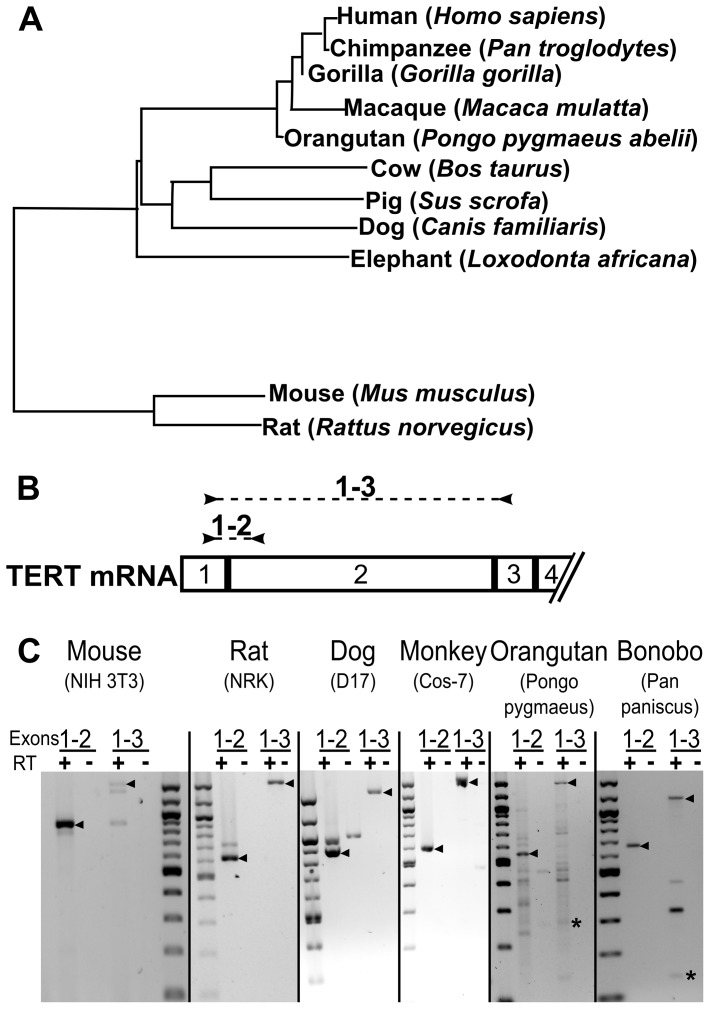
Exclusion of exon 2 from hTERT mRNA is specific to primates. A. A phylogenetic tree of TERT mRNA coding regions was created with the Jalview algorithm using percent identity neighbor joining. A genome sequence is currently unavailable for Bonobo, but is in the same genus (*Pan*) as chimpanzee. B. RT-PCR of TERT exons 1–3 of Monkey (Cos-7 cells), Mouse (NIH3T3), Rat (NRK), Dog (D17), Orangutan (*Pongo pygmaeus*) and Bonobo (*Pan paniscus*). An arrow indicates wild type bands and an asterisk indicates DEL[e2]. PCR reactions shown are representative of at least 2 trials.

To experimentally determine if exon 2 is excluded from TERT mRNA of other species, we obtained cell lines for mouse (NIH 3T3), rat (NRK), dog (D17) and African green monkey (Cos-7) and RNA from B lymphocytes of a bonobo monkey (*Pan paniscus*) and orangutan (*Pongo pygmaeus*). We prepared total cellular mRNA from each cell type and performed RT-PCR with primers spanning from exon 1 to exon 2 and from exon 1 to exon 3 (Figure 7B). In contrast to the human cell lines (Figure 2), we did not observe exclusion of TERT exon 2 from most of these species. However, primates, including the bonobo and orangutan, appeared to exclude TERT exon 2 from a fraction of their mRNAs (Figure 7C). We also observed a faint DEL[e2] band in some of the COS-7 monkey mRNA samples, but at a substantially reduced level compared to the human samples. This suggests that exclusion of exon 2 may be a phenomenon that is enriched in primates. Faint additional bands observed were either non-specific amplification products that were not derived from TERT mRNA sequences or were not consistently reproduced in experimental replicates.

We generated a phylogenetic tree of TERT mRNA coding regions of species that contain a large internal exon using the neighbor joining method (Figure 7A). This analysis is dependent upon the current genome annotations for each species, which may not be experimentally verified in all species. This phylogenetic analysis indicates that the TERT mRNAs of the primate species are much more similar to those of humans than the other species tested. This suggests that exclusion of hTERT exon 2 from mRNA transcripts may be a regulatory mechanism common to primates.

### 12 kDa polypeptide synthesized from an hTERT transcript that lacks exon 2

Translation of wild type hTERT begins in exon 1. Alternative splicing to exclude exon 2 generates a new reading frame in exon 3 with a premature termination codon. Translation of this mRNA is predicted to generate a polypeptide containing the first 73 amino acids of hTERT, encoded in exon 1, and an additional 23 amino acids encoded by exon 3, which are out of frame with the hTERT protein sequence. Thus, this mRNA variant would not be expected to generate a functional telomerase reverse transcriptase protein unless either a frameshift or a reinitiation event occurred in exon 3.

**Figure 8:**
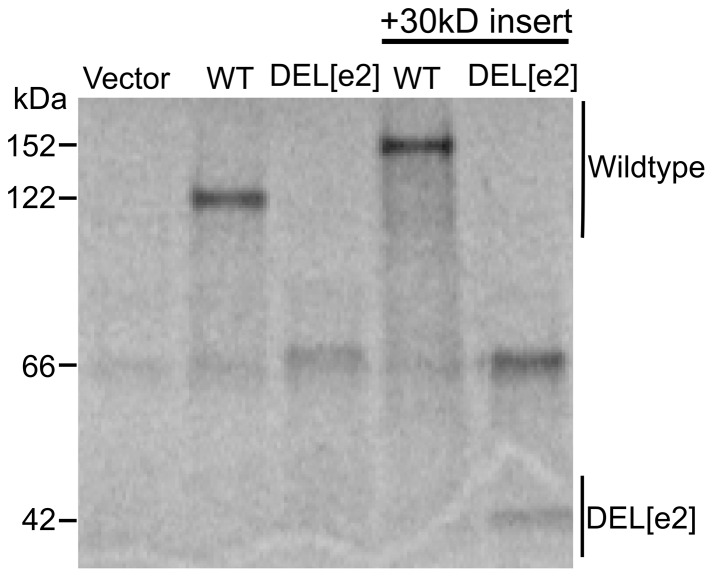
In vitro translation of hTERT mRNA lacking exon 2. Radiolabled in vitro transcription and translation of wildtype and DEL[e2] constructs in rabbit reticulocyte lysate. Wild type TERT produced the expected 122 kDa product, but a DEL[e2] product was not detected. Thus, sequences encoding 30 kDa of Upf3A were inserted into both constructs immediately after the hTERT start codon in exon 1. Protein products for wildtype (152 kDa) and DEL[e2] (42 kDa) were then detected. Products are separated by 4–20% gradient SDS-PAGE.

To analyze the proteins produced from a DEL[e2] mRNA transcript, we transcribed and translated hTERT and hTERT DEL[e2] plasmid DNA in a rabbit reticulocyte lysate. Although we were readily able to detect wildtype hTERT protein (122 kDa), the short DEL[e2] protein product (12 kDa) was not initially detected (Figure 8). This was possibly due to a lack of resolution of a 12 kDa polypeptide from the charged tRNA at the bottom of the gel. To enhance detection, we inserted DNA sequences encoding 30 kDa of protein immediately after the hTERT start codon in exon 1 of the hTERT and hTERT DEL[e2] constructs. After transcription and translation in vitro, we observed the expected protein species, enlarged by 30 kDa, derived either from the wild type (152 kDa) or DEL[e2] (42 kDa) mRNA transcripts (Figure 8). This supports our prediction that hTERT DEL[e2] generates a 12 kDa polypeptide, terminating in exon 3.

It is interesting to note that the full-length TERT protein from the insect *T. castaneum* has been characterized [33] and shown to lack the corresponding N-terminal domains encoded by exon 1 and most of exon 2 in mammalian TERT proteins. Thus, we asked whether the hTERT DEL[e2] mRNA might be reinitiated downstream to generate an active TERT. However, in this in vitro assay we did not observe either a reinitiation product or a frameshifting event from the hTERT DEL[e2] construct, which would allow production of the hTERT catalytic domains (Figure 8).

## Discussion

In humans, telomerase activity can be detected in actively dividing cells during embryonic development, as well as in adult germ-line tissues and tumors, but it is absent in many adult somatic tissues [12], [34], [35]. The ends of chromosomes in human cells that lack active telomerase progressively erode with each cell division. The level of cellular telomerase activity is predominantly regulated by transcription and alternative splicing of hTERT transcripts [36]. Alternative splicing allows for rapid reprogramming of gene expression patterns. The diversity and ratio of splicing regulators present determine the alternative splicing patterns [31], [32].

Examining splicing variants upstream of the hTERT reverse transcriptase domain is crucial because alternative splicing events that generate premature termination codons prevent translation of the downstream portions of hTERT. This is highlighted by the DEL[e2] hTERT variant, found in about half of all hTERT transcripts in A431 cells, which generates a premature termination codon within exon 3. Transcripts that lack exon 2 will generate a 96 amino acid polypeptide, mainly encoded by exon 1, regardless of any alternative splicing events which may occur in the downstream exons. Thus, well-studied downstream splicing variants, such as α (Figure 1), which is predicted to generate a dominant-negative protein [25], would not be produced from the DEL[e2] variant. The low ratio of exonic splicing enhancers/silencers in hTERT exon 2 may facilitate this splicing regulation.

Alternatively spliced variants of hTERT are also of interest because they may be responsible for secondary telomerase functions. Extra-telomeric functions of TERT have been reported that include WNT pathway signaling, mitochondrial localization, and regulation of oxidative damage-induced apoptosis [37]–[40]. Many of these functions do not require TR binding, or a functional TERT catalytic domain, and therefore make exciting possible functions for protein products of alternative hTERT mRNAs.

This study analyzed the relative steady-state levels of each detectable mRNA isoform. Steady-state levels of each species are influenced by their unique production and degradation rates. Almost all of the hTERT mRNA isoforms reported to date, including the deletion of exon 2 reported in this study, result in a frame shift [18]. This means that a premature termination codon is generated within the open reading frame. As a result, these mRNAs would likely become targets of NMD [19]. Thus, we predict that the values determined in this study may be an underrepresentation of the levels at which these alternative isoforms are actually transcribed. It would be interesting to analyze whether the level of hTERT mRNA isoforms is altered when NMD is inhibited by knocking down the central protein Upf1. If not, they may be resistant to NMD like retroviral RNAs [41].

Exclusion of TERT exon 2 appears to be an alternative splicing event acquired by primates. When we examined the alternative splicing of TERT in lower mammals, exon 2 was always included. However, in primate species, exon 2 was frequently excluded from TERT mRNA, although not always to the same extent as that observed for hTERT. This suggests that the DEL[e2] alternative splice variant may either be a consequence of the more complex cellular regulation, or it may represent a novel function of hTERT that is only present in primates. While we did not see alternative splicing of mouse exon 2 by RT-PCR, ESTs have been observed with partial deletions of mouse exon 2 that generate a frame-shift [42]. Thus, mice may have evolved a different type of alternative splicing involving exon 2.

It is worth noting that a catalytically active variant of hTERT protein could be generated from an mRNA that lacks exon 2 if translation started downstream of the wild type start codon. The functional elements minimally required for catalytic activity and TR binding are encoded by the nucleotide sequences in exons 3 through 16. While we were unable to detect such a protein product by in vitro translation, it is possible that translation of the DEL[e2] transcript could begin at an alternative start codon in vivo, which is in frame with the catalytic domains (amino acids 601–936) and the central region of the protein required for binding TR (amino acids 397–594) [43], [44].

The first 325 amino acids of hTERT, encoded by exon 1 and part of exon 2, encode for the hTERT TEN domain followed by a protein linker. The TEN domain is involved in low affinity TR interactions *in vitro* [45], [46] and sequence-specific recognition of single-stranded DNA [47]–[49]. Collins and colleagues have suggested that TERT lacking the TEN domain may be able to copy a long RNA template without trapping the template RNA-DNA hybrid in the TERT active site, thus preventing the processive telomeric repeat synthesis [50]. Consistent with this hypothesis, when the first 300 amino acids of hTERT are truncated, the resultant protein retains telomere repeat synthesis activity but generates predominantly short elongation products [51]. It was recently demonstrated that the human TEN domain (amino acids 1–325) can function in trans with the catalytic domain of hTERT (amino acids 326-end) to enhance telomeric repeat synthesis [50]. Further experiments will be required to determine if the 12 kDa protein derived from DEL[e2] transcripts plays any functional role in the cell.

## Materials and Methods

### Cell Culture

A431 (ATCC CRL-1555; epidermoid carcinoma), HeLa (ATCC CCL-2; cervical epithelial adenocarcinoma), HEK293 (ATCC CRL-1573; adenovirus 5 transformed embryonic kidney cells), NRK (ATCC CRL-6509; normal rat kidney epithelial cells), D17 (ATCC CCL-183; dog osteosarcoma lung metastasis) and COS-7 (ATCC CRL-1651; SV40 transformed African green monkey kidney fibroblast cells) cells were cultured in DMEM (Gibco) with 10% FBS and 1% antibiotics. HCT116 cells were cultured in McCoy's Medium (Gibco) with 10% FBS, 1% antibiotics, 2 mM glutamine and 1% NEAA. NIH3T3 cells were cultured in DMEM (Gibco) with 10% FCS and 1% antibiotics. Orangutan (*Pongo pygmaeus*; Coriell PR01052 peripheral vein biopsy, B-lymphocytes) and Bonobo (*Pan paniscus*; Coriell PR00748 peripheral vein biopsy, B-lymphocytes) RNA was generously donated by Sara Sawyer (University of Texas, Austin).

### Reverse transcription and RT-PCR

Total cell RNA was extracted using RNABee (Tel-Test). RNA was treated with DNAse I (Roche) for 20 min in 1x buffer (40 mM Tris-HCl, 10 mM NaCl, 6 mM MgCl_2_ 1 mM CaCl_2_; pH 7.9) followed by phenol extraction and ethanol precipitation. 1 µg of total RNA was used for oligo(dT) primed cDNA production using MMLV reverse transcriptase (Promega). 10% of the cDNA reaction was used in a two-step PCR reaction using Phusion DNA polymerase (Finnzyme). 30% of the PCR reaction was analyzed on a 2% agarose gel stained with ethidium bromide.

### Generation of hTERT competitive RNA

Primers flanking each desired deletion site were treated with T4 polynucleotide kinase (NEB) in 1x ligase buffer (50 mM Tris-HCl, 10 mM MgCl_2_, 1 mM ATP, 10 mM DTT; pH 7.5) for 1 hr. Primers were phenol extracted and precipitated, then used for inverse PCR to amplify the hTERT pGRN121 plasmid (Geron) except for the deleted sequence. The resultant PCR product was used for an intramolecular ligation. The five deletion sites were generated sequentially in the same plasmid. 1 µg of hTERT deletion DNA was linearized at the 3′ end of the open reading frame with XmaI and RNA was generated in vitro using T7 RNA polymerase in 1x reaction buffer (10 mM NaCl, 40 mM Tris-HCl, 6 mM MgCl_2_, 2 mM spermidine; pH 7.5). The RNA was polyadenylated using E. coli poly(A) polymerase (NEB).

### Competitive PCR

An adaptation of the protocol of Zentillin and Giacca (27) was used to quantitate TERT mRNA isomers by competitive PCR. A431 cells were trypsinized and counted using the Bio-Rad TC10 Automated Cell Counter. RNA was isolated using RNABee and treated with DNaseI as described above. 1 µg of total RNA was mixed with in vitro transcribed and polyadenylated competitive hTERT RNA in a two fold dilution series. cDNA was generated using 5 µM oligo(dT)_18_ and 1 µM random hexamers with 100 units of RNaseH-negative MMLV RT (Ambion) in 1x reaction buffer (0.5 mM dNTPs, 50 mM Tris, 75 mM KCl, 5 mM DTT, 3 mM MgCl_2_; pH 8.3). 10% of the cDNA reaction was used in a two-step PCR reaction using Phusion DNA polymerase (Finnzyme). Four identical PCR reactions were removed every two cycles, while the wildtype hTERT amplicon was empirically determined to be amplifying in the logarithmic phase. 5% of the PCR reaction was denatured in an equivalent volume of 8 M Urea loading dye and separated on a 4% denaturing Urea-acrylamide sequencing gel. The gel was dried under vacuum, imaged on a phosphorimager screen and analyzed using the ImageQuant software. The reaction in which the hTERT mRNA and competitor were present at equal amounts and amplifying at equivalent rates was used for quantification. The amount of hTERT mRNA variant per cell was calculated as the number of hTERT mRNAs relative to the known number of competitor molecules in the PCR reaction, divided by the number of A431 cells that were used to generate the cDNA. Calculations were performed as follows: [(isoform intensity/competitor intensity) (molecules of competitor added)]/(μg of RNA in RT reaction/average μg of RNA per cell).

### Conservation and RNA-seq analysis of TERT mRNA expression

120 human RNA-seq datasets were downloaded from the Sequence Read Archive database at NCBI; all had been sequenced on an Illumina platform. mRNAs were aligned to a custom Bowtie index [52] of large exon-containing genes [24], and reads per kilobase per million reads (RPKM) were calculated for each exon. Percent inclusion of exon 2 was determined by comparing its expression to that of the upstream and downstream exons, as described [24]. Conservation was calculated according to the Jalview and AMAS methods [53], [54].

### In vitro transcription and translation

Radiolabeled in vitro transcription and translation was performed with the TNT Quick-coupled Transcription/Translation Kit (Promega), using the pGRN121 wild type TERT cDNA plasmid (Geron). Sequences encoding hTERT exon 2 were deleted by inverse PCR. The reaction products containing [S^35^]-radiolabeled proteins were separated on a 4–20% precast gradient gel (Bio-Rad). Approximately 800 base-pairs of Upf3A DNA was inserted immediately downstream of the start codon of hTERT in order to increase the size of the protein products by 30 kDa.
